# Incorporating a Behavioral Medicine Approach in the Multi-Modal Management of Chronic Equine Gastric Ulcer Syndrome (EGUS): A Clinical Commentary

**DOI:** 10.3390/ani15203019

**Published:** 2025-10-17

**Authors:** Mary Klinck, Amy Lovett, Ben Sykes

**Affiliations:** 1 Mary Klinck, Veterinary Behavioural Medicine Consultant, Vaudreuil-Dorion, QC J7V 0K5, Canada; maryklinck@hotmail.com; 2 School of Agricultural, Environmental and Veterinary Sciences, Charles Sturt University, Wagga Wagga, NSW 2650, Australia; alovett@csu.edu.au; 3 BW Sykes Consultancy, Coffs Harbour, NSW 2450, Australia

**Keywords:** horse, stomach, equine squamous gastric disease, equine glandular gastric disease, problem behavior, pain

## Abstract

**Simple Summary:**

Equine gastric ulcer syndrome refers to a collection of stomach diseases in horses. Diseases of the lining of the stomach can be painful in affected horses. This in part explains why equine gastric ulcer syndrome continues to receive substantial attention in the equine medical, welfare and equitation research sectors. There is a complex interplay between EGUS and a variety of physical and psychological stressors. Affected horses are often presented to veterinarians with a history of problem behaviors, some of which resolve following treatment for gastric ulcers. However, problem behaviors persist in other cases, despite apparent (videoendoscopic) resolution of gastric ulcers. Some of these horses can have pain-related learnt, anticipatory behavior, even after the original source of pain has resolved. A behavioral medicine approach can benefit such cases, as well as chronic or refractory gastric ulcer cases. This includes the management of underlying disease(s), environmental modification, behavior modification, and, in select cases, behavior-modifying medication. This commentary, based on the authors’ clinical experiences and current literature, will explore how behavioral medicine can be integrated with traditional pharmacologic, nutraceutical, and husbandry strategies for the multi-modal management of gastric ulcers in horses, with a focus on managing the horse’s experience to improve case outcome.

**Abstract:**

Equine gastric ulcer syndrome (EGUS) refers to mucosal gastric disease in horses, including equine squamous gastric disease (ESGD) and equine glandular gastric disease (EGGD), which present as two distinct disease entities differing in pathophysiology and approach to disease management. Both diseases are a source of pain in affected horses, partly explaining why EGUS continues to receive substantial attention in the equine medical, welfare and equitation research sectors. There is a complex interplay between EGUS and a variety of physical and psychological stressors. Horses with EGUS are often presented to veterinarians with a history of problem behaviors, some of which resolve following gastroprotectant therapy. However, problem behaviors persist in some cases, despite gastroscopic resolution of disease. Some of these horses have pain-related learnt, anticipatory behavior, even after the original source of pain has resolved. Such cases, as well as chronic or refractory EGUS cases, can benefit from a behavioral medicine approach. This includes the management of any underlying diseases, environmental modification, behavior modification, and, in select cases, behavior-modifying medication. This commentary, based on the authors’ clinical experiences and current literature, explores how behavioral medicine can be integrated with traditional pharmacologic, nutraceutical, and husbandry strategies for the multi-modal management of EGUS, with a focus on managing the horse’s experience to improve case outcome.

## 1. Introduction

This clinical commentary, based on the authors’ clinical experiences and current literature, discusses the incorporation of veterinary behavioral medicine strategies as part of a comprehensive approach to long-term management of equine gastric ulcer syndrome (EGUS) in horses. This includes the application of behavior modification therapy for managing learnt, anticipatory behavior that develops because of pain. It aims to provide a framework for the practical implementation of such modification strategies, focusing as much on the impact of gastric disease on the horse as on the presence of disease per se.

## 2. Pain and Behavior

Animal pain has been described as “an aversive sensory and emotional experience representing an awareness by the animal of damage or threat to the integrity of its tissues”, which “changes the animal’s physiology and behavior to reduce or avoid damage, to reduce the likelihood of recurrence, and to promote recovery” [1]. Pain can have many effects on behavior; indeed, changes in behavior, including facial expressions, are often used to detect and assess pain in pre- or non-verbal humans, and animals [2,3,4], and various painful disease conditions have been reported as the source of problem behaviors in animals [5]. However, animal pain has historically been under-recognized [6], and pain-related behaviors are commonly misattributed to other causes, such as an individual’s temperament. In horses, pain can lead to problem behaviors during both unmounted handling and ridden work; it can also produce behavior changes that occur outside of interactions with humans [7]. Pain-based problem behaviors include avoidance and aggression during handling (e.g., “girthiness”), as well as resistance and altered responsiveness to previously learned cues under saddle.

## 3. Pain-Related Problem Behaviors in the Ridden Horse

The profession’s understanding of pain assessment during riding has been dramatically advanced by the substantial body of work validating the Ridden Horse Pain Ethogram (RHPE) [8]. This work has demonstrated that some problem behaviors under saddle in a trained horse can be clinical signs of musculoskeletal pain. A detailed discussion of this expansive work is beyond the scope of this review, but several critical elements warrant highlighting:

- Problem behaviors during ground handling and under saddle, historically often attributed to the horse’s personality, can often be attributable to pain when an appropriate, detailed medical investigation is performed [8,9].
- Such pain-based problem behavior can be reactionary or anticipatory. The role of anticipatory behavior has historically been overlooked, but horses with musculoskeletal disease display more abnormal behavior during tacking up than unaffected horses, suggesting the formation of complex anticipatory, pain-based behavior in some affected horses [10].
- Diagnostic analgesia of the primary disease condition improves RHPE scores but not completely, suggesting that learnt behavior can persist beyond the removal of the primary inciting cause of pain [11].

The RHPE was validated for the detection of equine musculoskeletal pain, but a recent study of horses with EGUS found a correlation between gastric disease scores and RHPE scores [12]. Work on the RHPE highlights the fact that nonspecific behavior problems historically attributed to temperament or primary behavioral processes (e.g., “attitude”, “naughtiness”, “knowing they can get away with it”) can actually indicate the presence of physical pain or discomfort in a trained horse.

## 4. Equine Discomfort Ethogram

The Equine Discomfort Ethogram (EDE) catalogues several postures, facial expressions, and other behaviors associated with pain or discomfort in horses [13]. This ethogram was developed based on review of video recordings of stalled horses with a variety of sources of pain. Several of the behaviors in this ethogram are reminiscent of some of the problem behaviors reported in EGUS, e.g., romping (alternately bucking and rearing), kicking out or back, kicking at the abdomen, backing, conservative movement, and uncharacteristic aggression and hyper-responsiveness [14]. It is likely that certain contexts or activities might increase or decrease the manifestations of behaviors perceived as problems; stalled horses have been found to decrease expression of pain or discomfort when people are present, which might be an evolved strategy in a prey species, to defend against predation [15].

It should be considered that different pain-based behaviors might be manifested in horses with EGUS when stalled or at liberty vs. when handled on the ground or ridden. Traditional horse training that involves negative reinforcement and positive punishment (see below) tends to inhibit behaviors. In horses handled on the ground, both the mere presence of a human and a history of training to stand still and tolerate various types of touch and manipulations might decrease or delay manifestations of pain due to EGUS, or alter the expression of these behaviors to those more likely to be tolerated by the handler (e.g., pinning of the ears and other facial expression changes, vs. biting or kicking). In ridden horses, pain might be manifested more strongly because movements and rider aids (e.g., leg pressure) might directly elicit a pain response, or because the horse is freer to express pain during riding than when handled on the ground. Critically, the above discussion highlights the disconnect between the handler or rider’s perspective and what is acceptable to them, and the horse’s perspective and its ability to express pain within the limits imposed on its behavior by humans. The focus of both the RHPE and EDE, and of this article, is to shift this perspective to a more horse-centric consideration of pain-based behavior problems, with increased consideration of the horse’s experience, more than the historical focus primarily on the handler or rider’s experience.

## 5. Clinical Signs of EGUS

Historically, appetite and weight loss changes have been considered the primary clinical signs of Equine Gastric Ulcer Syndrome (EGUS) [16]. This likely reflects the prominence of Equine Squamous Gastric Disease (ESGD) in the profession’s initial understanding of gastric disease and the relatively strong link between ESGD and changes in appetite [17,18]. However, pain-based problem behavior has increasingly been recognized as a primary presenting complaint for horses with EGUS [9,19]. This likely reflects an increased awareness of Equine Glandular Gastric Disease (EGGD), which is generally regarded to have a primarily behavioral presentation, and an overall increased focus within the profession on the relationship between pain and problem behavior [8,9]. The perceived association between EGUS and pain-based problem behavior is supported by a recent study reporting over 1400 gastroscopies in which owner-reported “bad attitude” or “girthiness” was the most common clinical sign associated with both ESGD and EGGD [14]. Behavior problems associated with EGUS can occur during unmounted interactions with people or other animals, as well as under saddle, and can include signs associated with tension (e.g., tail swishing, pinning the ears back), avoidance (e.g., evasion during tacking up, unwillingness to stand still during mounting, reluctance to move off the leg), and active resistance or aggression (e.g., biting, kicking, rearing, bucking).

There is a large body of research, accumulated over a 25-year-plus period, into pharmaceutical and nutraceutical interventions on the gastroscopic appearance of the stomach, but little attention has been paid to the impact of various interventions on the initial presenting clinical signs, e.g., behavioral changes. Two recent studies investigating the efficacy of extended-release injectable omeprazole demonstrated little or no change in behavior with endoscopic resolution of either ESGD or EGGD [20,21].

As discussed below, the reasons for this failure of the behavior to improve have not been investigated, but might include false attribution of the clinical signs to gastric disease when another cause (e.g., musculoskeletal disease) was concurrently present, incorrectly defining lesions as healed when ongoing gastric disease might still be present, the presence of ongoing or unresolved abnormal pain processing (e.g., central sensitization), and the potential impact of learnt, anticipatory behavior such that pain-based problem behaviors can persist even after the initial trigger is removed.

- The importance of addressing the potential for missed or incomplete diagnosis, or for different causes of pain to present similarly, whereby the need for comprehensive, holistic veterinary evaluation of horses presenting with problem behavior has recently been highlighted [9].
- Similarly, the challenges of assessing healing, especially in EGGD, have also been highlighted [22]. Significant inflammatory changes can be present within the glandular mucosa even when its gastroscopic appearance is normal [23]. Thus, although the gross appearance of the stomach might improve, ongoing gastritis might be present, making it difficult, if not impossible, for a treating clinician to be confident that true healing of the glandular mucosa has occurred, based on gastroscopic evaluation. The absence of expert consensus on what a “clinically significant” or healed EGGD lesion is [22] reflects this challenge. In light of this, the authors consider EGGD to be a disease continuum and manage it as such, in contrast to the dichotomous diseased/healed, abnormal/normal approach applied to ESGD.
- In addition, painful conditions such as EGUS might be associated with alterations in pain processing that persist beyond the resolution of the inciting injury or disease, resulting in ongoing hyperalgesia (increased perception of pain in response to a normally pain-producing stimulus) or allodynia (perception of pain in response to a normally nonpainful stimulus) as demonstrated in other species [24].
- Lastly, pain is a powerful teacher; it is associated with rapid learning modulated by fear [25]. It is a biological phenomenon that signals harm and emphasizes predictive learning of cues that signal pain [26]. Hence, a learnt, anticipatory behavior that persists beyond pain resolution can be present, as demonstrated by work performed using the RHPE [10].

## 6. Refractory EGUS

In addition to the clinical challenges above, refractory EGUS is common. Healing, defined by a return to ≤grade 1/4, occurs in only 77% of ESGD cases treated with omeprazole [27]. This healing rate decreases further when healing is defined as the restoration of the normal squamous mucosa (grade 0/4). Likewise, healing at 3 months for various EGGD therapies has recently been reported in a case series of over 3000 cases at 62%, regardless of the drug(s) selected (except for oral omeprazole monotherapy, which was inferior to the other therapies chosen (oral omeprazole and sucralfate, misoprostol, and extended-release injectable omeprazole) [28]. This means that pharmaceutically refractory disease is common, yet to date, little attention has been paid to managing such cases and the behavioral impacts of refractory disease.

## 7. The Role of Stress in EGGD

In considering the potential role of stress in EGUS, it is worthwhile to keep in mind the two-way interaction of stress and disease; i.e., stress can act as a potential driver of disease and disease can also act as a potential driver of stress, ultimately resulting in bi-directional effects and feedback loops [29,30].

Stress is widely recognized as an important contributor to EGGD [31,32]. Horses with EGGD have an exaggerated cortisol response to adrenocorticotropic hormone stimulation [32], whether this is cause or effect is not known. Pain can cause stress, and a recent report demonstrated that horses with concurrent musculoskeletal disease are more likely to be refractory to pharmaceutical treatment for EGGD [28], supporting the potential role of pain as a driver of gastric disease. Actively competing increases the risk of EGGD by more than 10-fold, lending support to the role of behavioral stress in gastric disease [33]. These findings highlight the need to manage behavioral stress and concurrent painful diseases as part of the long-term preventative and management strategies for EGGD in horses. The role of stress in the pathogenesis of ESGD is less clear. Changes in hair cortisol concentrations have been associated with the presence of ESGD [34], but whether the changes are cause or effect is not known.

Stress also plays an important role in nociception. In humans, chronic pain is associated with anxiety, depression, and catastrophizing, which further sensitizes the nervous system to pain [35]. Patients with chronic pain often exhibit heightened stress responses, such as decreased heart rate variability and exaggerated cortisol reactivity [36]. As demonstrated in other species, stress influences chronic pain through physiological, neurological, and psychological mechanisms, including:

- Hypothalamic–Pituitary–Adrenal (HPA) Axis Dysregulation: Chronic stress leads to prolonged HPA axis activation, resulting in increased cortisol production. Over time, this dysregulation can contribute to central sensitization, where the nervous system becomes hypersensitive to pain stimuli [24].
- Inflammation and immune system activation: Stress-induced activation of the immune system increases pro-inflammatory cytokines, such as IL-6 and TNF-α, which enhance pain signaling in the nervous system [37].
- Neural plasticity and central sensitization: Prolonged stress alters brain regions associated with pain processing, including the amygdala, anterior cingulate cortex, and prefrontal cortex, leading to exaggerated pain responses [38].

## 8. Sleep Deprivation

Although most rest and sleep in horses are done standing, REM sleep involves a loss of muscle tone and, as such, requires recumbency. Horses need approximately 20–40 min of REM sleep per day; when they cannot lie down to rest, they cannot engage in REM sleep, and consequently become sleep-deprived [39,40]. The classic clinical sign of sleep deprivation in horses is loss of consciousness with falling from a standing position or beginning to fall and catching themselves. However, this sign might not always be observed. Other signs include standing sleep in which the horse assumes an unnaturally low head posture or in which the horse’s body sways [41]. In the absence of observation of these signs, the owner might note secondary effects, such as abrasions on the dorsal aspect of the front fetlocks, or on other parts of the body (injuries sustained as the horse’s legs buckle and it begins to fall).

It is well-recognized in human medicine that sleep deprivation is an important risk factor for psychological and physical health ailments [42], and that it is associated with alterations in pain processing and hyperalgesic states [43]. In turn, pain and discomfort are known to interfere with sleep in other species [44]. The potential for horses with EGUS to have subtle behavioral changes that are only expressed when the caretaker is absent, but that might be associated with sleep deprivation, has recently been demonstrated [7]. As prey animals, horses require a sense of safety in order to lie down to sleep, and anxiety associated with pain can interfere with their willingness to lie down [45]. Since pain, discomfort, and anxiety can contribute to sleep deprivation, and sleep deprivation itself induces physiological stress, it follows that EGUS could produce sleep deprivation as a sequela, and that sleep deprivation for any other reason could contribute to EGUS.

Sleep deprivation deleteriously affects learning in horses [39] and other species [46,47]. Likewise, pain negatively impacts cognition in humans, affecting attentional, executive and general cognitive functioning [48]. There is potential for this to result in a self-perpetuating loop of behavioral stress for horses wherein increased stress is experienced during training due to impaired cognitive capacity triggered by either pain or sleep deprivation; this increased stress alters sleep, and in turn, altered sleep decreases learning capacity, leading to increased stress during training. The impact of such a cycle might be exacerbated in specific disciplines, e.g., greater emotional responses have been reported in dressage horses than in other equine athletes [49], which might suggest increased susceptibility to such added stresses.

Sleep deprivation also influences emotions and associated behavioral responses. The amygdala has an important role in the emotional response to aversive stimuli, and the prefrontal cortex exerts a descending inhibitory effect on these responses. In humans, sleep deprivation has been found to produce an amplified, hyper-limbic response to aversive stimuli, likely associated with decreased inhibitory control from the prefrontal cortex [50].

## 9. Behavior Modification Therapy (BMT)

Behavior modification therapy is a cornerstone of chronic pain management in human medicine [51]. This is in direct contrast to the lack of attention paid to managing the behavioral aspects of chronic, painful diseases in the horse. Commonly used techniques in human medicine include: cognitive behavioral therapy (CBT) to help patients identify and change negative thought patterns related to pain [52], mindfulness and relaxation techniques (e.g., meditation) to lessen the emotional reaction to pain [53], and behavioral activation to encourage patients to engage in enjoyable activities despite pain [54]. In addition, pain-related fear in humans can be treated with extinction (repeated exposure to the stimulus that predicts pain, without the experience of pain) or counterconditioning (pairing the stimulus predicting the pain with another stimulus that generates a positive emotional response; see below) [26].

Evidently, cognitive processes are a focus of some of the human treatments for pain. Animal cases cannot be treated in this way; hence, the omission of the term “cognitive” from veterinary and non-veterinary animal behavior therapy. However, even without being able to have animals consciously participate in this way, there are potential applications of these types of treatments to animals. Whether such therapies can benefit horses with chronic painful diseases, such as EGUS, is largely unexplored. However, counterconditioning and desensitization are commonly recommended for the management of fear of procedural pain and discomfort in animals, and recent studies have examined the efficacy of this technique for veterinary examination in dogs [55,56], claw-trimming in cats [57], and various husbandry, examination, and injection procedures in horses [58]. In addition, the American Animal Hospital Association’s most recent canine and feline pain management guidelines specifically note the beneficial influence of positive environments and emotions on pain, and include recommendations that might have similar effects to some CBT therapies used in human pain (e.g., behavioral activation), via environmental modification to provide easier access to preferred locations (e.g., using steps, ramps), as well as hiding places in the home, and both avoiding unpleasant interactions and providing opportunities to engage in preferred activities and interactions [59].

## 10. Behavioral Medicine

In veterinary behavioral medicine, the approach to behavioral disease or problem management involves the following: (1) management of any underlying contributory physical disease(s); (2) environmental modification to reduce stress and anxiety in general, as well as to control exposure to specific stimuli associated with the problem behavior, and to maximize safety; (3) behavior modification techniques to change the animal’s emotional and behavioral responses in the problem contexts; and, 4) behavior modifying medications, as needed, to reduce anxiety, fear, and impulsivity.

### 10.1. Management of Underlying Disease(s)

The importance of a holistic veterinary approach when investigating pain-based problem behavior in horses has recently been highlighted [9]. Specifically, clinicians are cautioned against common clinical biases such as anchoring bias, specialist bias, and confirmation bias, all of which are common in clinical medicine [9] and can lead to an inaccurate or incomplete diagnosis. Further, clinicians are encouraged to consider the potential for multiple concurrent painful diseases to be present [9]. For instance, in competition horses, both EGUS and musculoskeletal disease are highly prevalent, and thus, are likely to be concurrent co-contributors to pain-based problem behavior [9]. Untreated concurrent musculoskeletal disease could contribute to a perceived poor clinical response to EGUS treatment (i.e., problem behavior persisting despite EGUS treatment), as well as to recurring disease.

It is also important to recognize that not all diseases can be cured, and that management of ongoing disease is important. An essential element of this is removing the nociceptive trigger during peak at-risk periods to reduce disease impact and consequent pain-based problem behavior. With specific reference to EGUS, before and during exercise are the most common owner-reported trigger periods for problem behavior. The authors’ approach to reducing the nociceptive trigger during exercise depends on the underlying disease. For EGGD, it includes optimizing mucosal barrier protection with targeted, pre-exercise sucralfate and ongoing supplementation with appropriate nutraceuticals [60]. Additionally, pre-exercise buffering with marine-derived calcium carbonate supplementation [61] is anecdotally beneficial. Targeted pharmaceuticals can also be used to reduce the total acid load in the stomach, although the authors try to reserve these for competition periods only. A similar approach is used for ESGD by the authors, with a greater focus on reducing the total acid load in the stomach. In addition to the aforementioned approaches, targeted pre-exercise feeding of hay, especially alfalfa, is recommended for this purpose.

### 10.2. Environmental Modification

Environmental modification for the behavioral treatment of EGUS is comparable to that for other behavior problems, in that it begins with meeting the horse’s behavioral needs, in particular by providing the three “F”s (3Fs): friends, freedom, and forage [62]. This is expected to have both direct and indirect benefits—e.g., insufficient access to forage, and confinement, in particular, are known risk factors for ESGD—and the 3Fs also provide behavioral benefits by reducing stress and the behavioral impact of disease on a horse with EGUS. In addition, positive cognitive bias, or optimism, is known to have a protective effect in human pain [63], and research to date has found that horses living in more natural conditions with respect to space, forage, and social interactions have responses more consistent with optimism than do horses in more restricted environments [64,65].

Horses evolved to live in relatively stable social groups, and to spend most of their day foraging and eating. As a prey animal, group living provides security in the form of shared vigilance and defense from threats [66]. However, changes in social group composition and housing with incompatible individuals can be a source of stress. Thus, in applying environmental modification for any given horse, not only housing with other horses, but the nature of the group and its members’ interactions, the space available to permit individuals to choose to interact or to avoid other group members, and the stability of the group, should all be evaluated and optimized. In addition to turnout, stable design plays an important role in the social environment of horses that are confined for part of the time; there are benefits associated with visual and tactile contact between stabled horses [67], as well as during turnout.

Ad libitum access to roughage is widely recommended for the prevention of ESGD [16]. It is also behaviorally important for horses, which have a strong motivation to spend most of their time foraging. Thus, consumption of daily calories in a condensed timeframe can lead to a behavioral vacuum, frustration and boredom. In a so-called “natural” environment, the sources of vegetation would be varied, and the horse would devote time and attention to selecting particular forages [66]. This is often not the case in the domestic environment, but opportunities to replicate it can be provided (e.g., providing varied types of forage, using different types of feeders, ground-feeding loose forage, or using toys or hay nets to increase foraging time and effort).

“Freedom” in the form of turnout is important for more than just the provision of exercise; it provides increased opportunities for exploration and for making choices. Stall confinement has been found to be associated with the development of ESGD [68], as well as other signs of stress, such as stereotypies, aggressive behavior, and lack of interest in the environment [69]. In addressing environmental modification, it is important to consider the precise circumstances of each case, and the size, layout, and complexity of the environment, because “turnout” can have different meanings, e.g., a horse on a 40-acre pasture clearly has more opportunities for exercise and foraging, and more ability to control its social interactions with groupmates, than a horse in a ¼ acre sand paddock, even though both situations are considered “turnout”.

In addition to the 3Fs, factors such as amount and type of work performed by the horse, training and handling methods, predictability of the daily routine and environment, and other particularities of the environment (e.g., any visual or auditory stimuli to which the horse reacts fearfully and does not habituate), can all influence general stress levels. For instance, an association has been found between a greater number of riders and caretakers and an increased risk of EGGD [70]. Thus, in each case, it is appropriate for a review of the horse’s management to be performed so that modifications can be implemented both to eliminate or reduce stressors, and to fulfill behavioral needs [71,72].

### 10.3. Behavior Modification

Pharmacologic treatment of the underlying physical disease and implementing a general stress reduction via environmental modification strategies, as described above, can improve if not resolve behavior problems resulting from EGUS. However, pain-based problem behavior can persist because of learnt, anticipatory responses. In such cases, behavior modification in the form of specific training might be needed to change horse behaviors that have developed in response to EGUS.

#### 10.3.1. Learning Theory

Two broad categories of learning that are commonly used in behavior modification are classical conditioning and operant conditioning.

**Figure 1:**
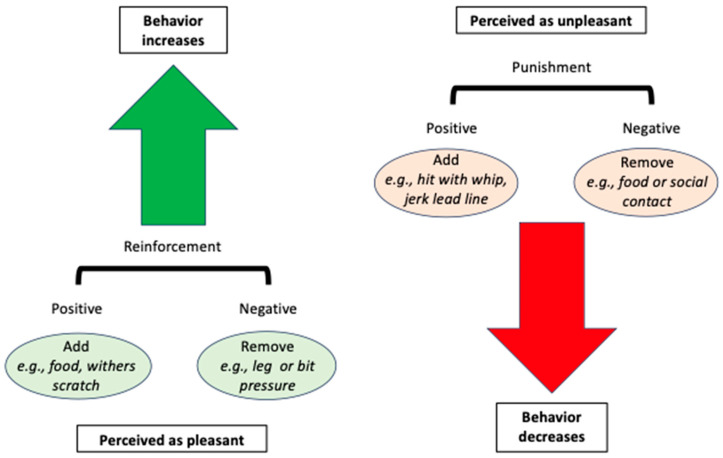
A schematic of operant conditioning for behavior modification of horses. Reinforcement (green arrow) is perceived as pleasant and increases the future likelihood of a behavior. It can be positive (something pleasant is added, such as food), or negative (something unpleasant is removed, such as leg pressure). Punishment (red arrow) is perceived as unpleasant and decreases the future likelihood of a behavior. It can be positive (something unpleasant is added, such as a strike with a whip), or negative (something pleasant is removed, such as food or social contact).

- In classical, or Pavlovian, conditioning, an unconditioned stimulus (UCS) is associated with an unconditioned response (UCR). When a neutral stimulus (NS) is presented just before the UCS, the NS becomes a conditioned stimulus (CS) that generates the previously UCR: now the conditioned response (CR). The classic example of this is Pavlov’s experiment: a bell was rung (NS), and then food was presented (UCS), and the dogs salivated (UCR). After several repetitions, the sound of the bell became a CS that produced a CR (salivation).
  ○ An example of classical conditioning would be a horse that experiences pain during girthing (Figure 1). Initially, the UCS is pain as the girth is tightened, and the UCR is the associated emotional response of fear and distress. The NS is the girth being fastened or manipulated, i.e., the step in the sequence that directly precedes the pain, fear, and distress. Over time, the NS becomes a CS that produces a CR of fear and distress. Additionally, neutral stimuli that precede the CS can become new conditioned stimuli, and the cycle can repeat so that the CS for the CR of fear and distress can move earlier and earlier in the sequence, such that the horse reacts when it is approached with the saddle. This process can also associate pain experienced during riding with being tacked up.
- In operant conditioning, a behavior results in a consequence, which can either increase or decrease the future likelihood of the behavior. Reinforcement indicates that the consequence increases the likelihood of the behavior, and punishment indicates that the consequence decreases the likelihood of the behavior. What constitutes reinforcement vs. punishment is therefore based entirely on the animal’s behavior, not on the handler’s expectation of what will happen, nor on the handler’s perception of what should be pleasant or unpleasant for the animal. Additionally, consequences can arise that are not delivered or controlled by the trainer. Reinforcement and punishment can either be positive or negative. “Positive” indicates that something is added, while “negative” indicates that something is removed.
  - Positive reinforcement means adding something (e.g., a food reward, scratching the withers) that the horse desires or enjoys enough to increase the future likelihood of the preceding behavior;
  - Positive punishment means adding something (e.g., being struck with a whip, acute EGUS pain) the horse wishes to avoid enough to decrease the future likelihood of the preceding behavior;
  - Negative reinforcement means removing something (e.g., leg or bit pressure) the horse wishes to avoid enough for this removal to increase the future likelihood of the preceding behavior;
  - Negative punishment means removing something (e.g., access to feed or social contact) the horse desires enough for this removal to decrease the future likelihood of the preceding behavior.

#### 10.3.2. Secondary Reinforcer

**Figure 2:**
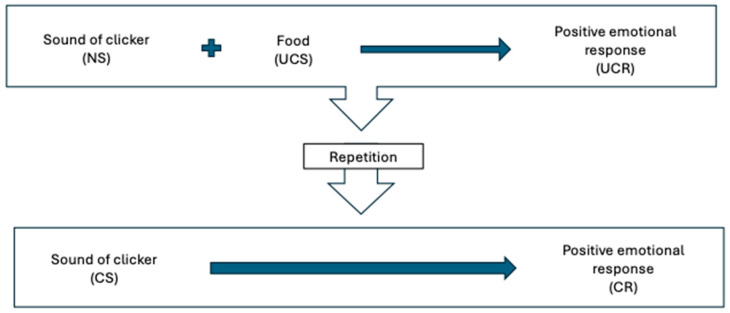
Classically conditioning a clicker as a secondary reinforcer. The clicker, a neutral stimulus, is paired with food, an unconditioned stimulus that generates an unconditioned response (positive emotional response to food). After repeated pairings, the clicker becomes classically conditioned (conditioned stimulus) to predict the food and associated positive emotional response (conditioned response). NS—neutral stimulus; UCS—unconditioned stimulus; UCR—unconditioned response; CS—conditioned stimulus; CR—conditioned response.

Timing is important for creating associations: the more closely associated in time or space two stimuli are (classical conditioning) or a behavior and its consequence are (operant conditioning), the more likely it is that a learned association will develop. Because it is sometimes difficult or impossible to give a reward during or immediately after the desired behavior, a secondary reinforcer (also called a conditioned reinforcer or bridging stimulus) is often used. This is the principle behind clicker training. A secondary reinforcer is a classically conditioned stimulus that is used to signal to the animal that the primary reinforcer (e.g., food) is coming. It can be a click, or any other distinctive and consistent sound, even a word always said with the same intonation, such as “good”, “yes”, or a non-auditory stimulus. See Figure 2.

**Figure 3:**
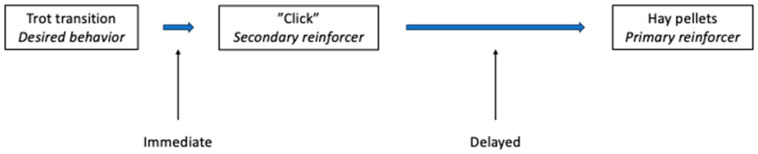
Use of a secondary reinforcer to mark the desired behavior, during ridden work, permits delivery of the primary reinforcer after a short delay. After classically conditioning the clicker as a secondary reinforcer, it can be used in operant conditioning to positively reinforce a desired behavior. Thus, in retraining an upward transition to trot, as soon as the horse trots, the clicker is sounded (secondary reinforcer), indicating that food (the primary reinforcer) is coming. The sound of the click produces the positive emotional response just as the desired behavior (trot transition) is performed. This is expected to increase the future likelihood of the trot transition in this context. Food can then be delivered after a short delay, i.e., the time it takes for the horse to stop and the rider to deliver, e.g., hay pellets.

After a secondary reinforcer is classically conditioned, it can be used to indicate to the horse the precise moment when it is performing the desired behavior (e.g., during the transition to trot from walk) and then the primary reinforcer (e.g., food) is delivered just afterwards. For a secondary reinforcer to maintain its reinforcing properties, it must always be followed by the primary reinforcer; in addition, although there can be a delay between the secondary reinforcer and the primary reinforcer, this should be kept as short as possible (e.g., saying “good” as the horse initiates trot, then allowing the horse to stop, and delivering of food). See Figure 3.

#### 10.3.3. Applying Learning Theory to Pain-Based Problem Behavior

Traditionally, horse training has used discomfort and other unpleasant experiences, in the context of negative reinforcement (e.g., releasing leg pressure in response to the horse executing a correct movement) and positive punishment (e.g., striking the horse, or tugging on its bit, in response to undesirable behavior), to modify behavior. While these methods can be effective in the basic training of horses, it is essential to recognize that their use can be counterproductive, especially for behaviors motivated by fear, anxiety, or pain. This is because both negative reinforcement and positive punishment involve aversive experiences (i.e., doing something that the horse finds unpleasant), that can increase fear and anxiety.

Instead, addressing pain-based problem behavior should focus on using positive reinforcement techniques to change the emotional (classical conditioning) and behavioral (operant conditioning) responses. In practice, while the emphasis might be on one or the other, emotional and behavioral responses will both be affected by any training.

#### 10.3.4. Systematic Desensitization and Counterconditioning

Changing the problematic emotional response and consequent pain-based problem behavior involves systematic desensitization and counterconditioning (DS/CC) to the associated stimuli. Successive approximations are used to reintroduce the problematic stimuli, and over several sessions, the stimulus intensity is gradually increased. Stimuli are then combined in a stepwise manner (still paired with something evoking a positive emotional response). If the horse reacts with tension, evasion, or aggression, the stimulus intensity is decreased to an earlier step in the process and the progression is delayed until the horse is relaxed [58].

Scoring tools for monitoring a horse’s state of relaxation vs. arousal are available (e.g., The Spectrum of Equine Fear, Anxiety, and Stress; Fear Free^TM^) [73]; changes in body language and facial expression, and decreased ease of handling and compliance with cues can all signal that a horse is moving out of a state of relaxation and that the training should be paused or moved back to an easier level. The objective of DS/CC is to change the emotional response of anxiety and fear (in this context, secondary to pain) to a relaxed and positive emotional state. Because reinforcement is provided in response to calm behavior, a behavioral response is also trained. It is important to set up training sessions for success by minimizing any other aspects of the environment that might cause agitation or distress (e.g., avoid training during feeding times, when other horses are being turned in or out, or when there are loud noises or frightening objects or activities in the vicinity).

Further, it is important to consider that anticipatory responses to pain are often context-specific and associated with a constellation of stimuli (e.g., rider, time of day, physical location, and the sequence of interactions), not just the obvious stimuli that trigger the overt pain-based problem behavior (e.g., tightening of the girth, or rider aids). Hence, retraining involves separating out the individual stimuli as much as possible, reducing the stimuli’s intensity to a level where the horse does not react, and pairing the individual stimuli with something the horse finds pleasant (e.g., food, or scratching in mutual grooming sites).

#### 10.3.5. Retraining Non-Ridden Behavior

A detailed description of the authors’ suggested retraining procedure for a common behavior problem encountered in ground handling, “girthiness”, is given in Table S1 as an example in the Supplementary Material. While the example might seem tedious, it is important to proceed very gradually when performing DS/CC because the horse needs to be kept below its threshold for reaction. It is more difficult to change an existing emotional reaction from a negative to a positive one than to train a positive emotional response to something completely new, and this translates to more time and more steps for retraining than for initial training. If the training steps are taken too fast and the horse goes over its threshold and reacts with evasion or aggression, it can be necessary to start over at the beginning.

Once the horse is comfortable proceeding through all the steps, it might be possible to transition to offering hay in a net while grooming/tacking up, to maintain the general association between a pleasurable stimulus (food) and the previously problematic interaction. This also accomplishes the objective of pre-exercise feeding of forage for the prevention of ESGD. However, the provision of free-choice hay during the problematic interaction should not replace a systematic DS/CC program.

#### 10.3.6. Retraining Ridden Behavior

As with the approach for pain-based problem behaviors during unridden handling, planning a retraining program for problem behaviors under saddle begins with identification of the problematic cues, as well as other aspects of the contexts in which the cues are given, the details of the problem behavior manifested by the horse, and clearly defining the desired responses. This process should also identify any signs leading up to overt problem behaviors (e.g., stiffening, putting the ears back, raising the head, mouthing the bit). In some cases, the stimulus that predicts pain might not be a rider cue but something else in the environment (e.g., moving towards a jump). Problem behavior shown during riding can be complex, involving many associated contextual stimuli. To address this, it can be helpful to reintroduce the desired behavior in a different context, e.g., on the lunge line, in hand, or in other contexts such as on trail rides.

The authors’ approach to retraining the association is like that described for ground behavior above and in Table S1 (Supplementary Material), except that instead of the horse being asked to tolerate handling, it is asked to perform an active behavior. For an example of the authors’ approach to retraining ridden behavior see Table S2 in the Supplementary Material. Therefore, there are added elements of ensuring that the horse “knows the answer to the question being asked” (i.e., the cue given clearly signals to the horse exactly what behavior is desired of it) and that it can willingly comply (i.e., it is physically capable of the desired response). Thus, retraining should begin at a level of effort and complexity well below that at which the horse had been working. This ensures the creation of opportunities for the horse to complete the requested task as easily as possible. Pain-free, successful repetitions paired with positive reinforcement increases the horse’s confidence and positive emotional associations with the desired task. The potential impacts of disease (e.g., via pain, stress and/or sleep deprivation) on cognitive capacity and the horse’s ability to respond favorably must also be considered; it is important to recognise that the horse’s capacity for or rate of learning might not be the same as before the disease.

The reintroduction of cues and target behaviors can then be planned. First, the cues and target responses are separated out from other stimuli associated with the context as much as possible. The location, people, other horses, or objects present (e.g., obstacles, jumps, or specific types of these), and time of day all need to be considered. Next, the problem sequence is broken down into steps, from before the start of the horse’s reaction signalling fear or anxiety, to after completion of the target behavior. Because both the cue and the target behavior itself can be associated with pain and fear, separating the desired behavior from its cue and reintroducing it with a different cue is helpful. The cue that was associated with the problem behavior can be reintroduced later, once the horse is comfortable performing the target behavior in response to the new cue. The behavior is then reintroduced incrementally, via successive approximations, and the horse is rewarded for success at each step. Once the target behavior has been reacquired, the desired cue is gradually reintroduced, and the other environmental stimuli that were separated from the target behavior are gradually reincorporated, in a stepwise manner, while continuing to reinforce the target behavior. If a horse reacts with tension, evasion, or overt fight-or-flight-type behaviours (e.g., bucking, rearing, bolting) during retraining, it is important to go back one or more steps in the retraining process. Moving on to the next step only occurs when the horse remains relaxed for several repetitions. Multiple sessions are expected to be required to achieve the previous level of response.

For behaviors under saddle or involving the horse in motion (i.e., where a primary reinforcer cannot be delivered during the desired behavior), the use of a secondary reinforcer such as a clicker is particularly helpful. This is used to signal to the horse at the exact moment that it is performing the desired action, and allows a small delay before delivering the reward, while maintaining the association between the reward and the desired behavior. After the secondary reinforcer is given (e.g., “click”), the horse is allowed to stop and is then fed a treat (e.g., hay pellets). While other reinforcers than food can be effective (e.g., withers scratching), food is likely to be the most consistently effective reinforcer for most horses. Patting or slapping the neck is not rewarding for horses [74,75].

#### 10.3.7. Adjunctive Therapies

Multiple studies have reported benefits of playing music for horses, including increased relaxation [76], decreased stereotypic and agitated behavior [76], decreased sentient and increased eating behavior [77], decreased salivary cortisol [78,79], and increased serotonin concentration [80]. The effects of music are cumulative: playing relaxing music for 3 h a day produced more positive effects on horses’ emotional state than playing it for 1 h [79]. Music type is important, with low to medium tempo music (particular types of classical and country music) resulting in more restful behaviours than jazz or rock [81]. Conversely, playing talk radio is associated with an increased risk of ESGD [82].

Massage might also be beneficial in some horses, though it is important to monitor response; horses with heightened pain processing might experience normally nonpainful touch as painful. The beneficial effects of massage are rapid, with multiple studies demonstrating a more relaxed state in horses, as evidenced by a decrease in heart rate, an increase in occurrence and duration of relaxation-related behaviors, and reduced salivary cortisol, in a single session [71,72]. Similarly, pupil size, a marker of sympathetic tone and stress response, decreases with a single massage intervention [83]. However, the effects are cumulative, with daily massage more effective at reducing heart rate, improving heart rate variability, and decreasing salivary cortisol, than massage once approximately every 3 weeks [79]. Horses receiving Swedish massage twice a week for 5 weeks had lower heart rates and improved flexibility of the horses’ necks, backs and shoulders [84], and thrice-weekly massage reduced pre- and post-exercise salivary cortisol over a 6-month period [78]. Massage has been shown to increase nociceptive threshold [85], and in addition to reducing stress, might have direct benefits in the management of pain-related problem behavior by improving pain threshold. Similarly, massage might have direct benefits on performance, with endurance horses receiving thrice-weekly massages winning more races and prize money than control horses in one study [78].

### 10.4. Behavior-Modifying Medication

The decision to prescribe medication to treat anxiety and fear-related behavior problems in a horse with EGUS-associated pain-based problem behavior depends on several factors. As a general rule, such medications are prescribed when an animal: (1) has an inherent tendency to anxiety, fear, or reactivity; (2) has unavoidable stressors in its environment or routine, with which it is having difficulty coping; or (3) has had (an) experience(s) that was (were) traumatic or stressful and is having difficulty recovering, despite removal of the stressors themselves.

Horses in categories 1 and 2 in particular, can be more reactive due to a generally increased level of arousal. In addition, in diseases where stress is thought to be a predisposing factor, such as EGGD, underlying anxiety, arising either from the individual’s temperament or from unavoidable environmental factors, might predispose the horse to the development and recurrence of EGGD. In such cases, behavior-modifying medication might be indicated. However, because it is common for horses to be managed in ways that increase their general level of stress, best practice is always to address the environment first, to remove avoidable stressors, as discussed in the Section 10.2, above.

As for category 3, in the authors’ clinical experience, some horses, particularly if they have experienced severe or repeated EGUS pain, can demonstrate anticipatory, fear-based behavior associated with handling and ridden work. Any unpleasant interactions with humans, such as punishment (“correction”) administered because of the problem behavior, only increases the horse’s fear and anxiety. If there is an inadequate response to positive reinforcement-based retraining attempts, or if a starting point for retraining at which the horse remains relaxed cannot be identified due to the intensity of its reactions, behavior-modifying medication is also indicated.

In EGUS cases with refractory pain-based behavior problems, the objective of behavioral pharmacological treatment is to reduce the horse’s level of anxiety and fear associated with the problematic interactions to facilitate behavior modification, i.e., the learning of new emotional and behavioral responses. In category 1 and 2 horses, an additional objective is to decrease general levels of anxiety, fear, and reactivity. The choice of behavior-modifying medication is influenced by the details of the problem behavior and individual horse health particularities. In cases for which a short series of discrete retraining sessions is envisioned, treatment with a rapid-onset, short-acting, “event” medication on an as-needed basis can be effective. In cases in which the problem behaviors appear in multiple contexts or are frequent or difficult to avoid, or in which retraining is expected to proceed over a longer period, medication given continuously over a longer period is likely to be more appropriate. In some cases, both types of medications might need to be used together. Information on behavior-modifying drugs is summarized in Table 1.

#### 10.4.1. General Considerations Regarding Behavior-Modifying Medications

There has been relatively little study of behavior-modifying medications in horses, but a detailed review of currently available pharmacological therapy for behavior management in horses has recently been published [86]. Most are not licensed for this use and hence are administered to horses off-label. In addition, most are not permitted in equestrian sports; treating veterinarians and owners should verify with the pertinent governing body the legality of any medications prescribed prior to participation in competition. It is also important to note that these medications will be ineffective, or incompletely effective, if the underlying painful disease that precipitated the problem behavior persists.

Any medication that affects behavior can have unforeseen effects on it. Hence, the horse should be monitored for both physical and behavioral side effects. Although not specifically described in horses, when serotonergic drugs are used in other species, and especially if they are combined with other medications, there is a risk of serotonin syndrome, a potentially life-threatening condition. The signs observed in other species include those associated with neuromuscular excitation (tremors, hyperreflexia, myoclonus, seizures), changes in mentation (confusion, restlessness, agitation, hyperactivity, hyperreactivity), autonomic dysregulation (changes in body temperature, mydriasis, tachycardia), and gastrointestinal derangements (anorexia, diarrhea, hypersalivation) [87,88].

For safety reasons, medications that might cause sedation should be avoided during ridden or driven work, and in any other context in which an altered state of consciousness or impaired balance or coordination could be dangerous. Further, moderate to heavy sedation is undesirable when performing behavior modification exercises, even on the ground, because it can impair learning. In addition, there is a risk that if the degree of sedation doesn’t prevent awareness but impairs behavioral expression (and human detection) of anxiety and fear, the horse might experience undetected anxiety or fear during training, with resulting worsening of the behavior problem.

#### 10.4.2. Delayed Onset, Long Duration Medications

Antidepressant-type medications such as tricyclic antidepressants (TCAs) and selective serotonin reuptake inhibitors (SSRIs) are commonly used to treat anxiety disorders in dogs and cats. Medications of this class are given daily and take 4–6 weeks to reach their full therapeutic effect. They are typically used for months to years; in some cases, treatment is lifelong. Assuming the medication is well-tolerated and effective, the minimum treatment duration is 4–6 months. If the medication is discontinued, the dose should be gradually tapered over a period of 4–6 weeks or longer to reduce the risk of rebound anxiety.

Fluoxetine, an SSRI, is the only drug of this type to have been studied for long-term use in treating anxiety-related disorders in horses [89]. The reported oral dose is 0.15–0.5 mg/kg q24-h [89,90]. One study showed subjective therapeutic benefit in 70% of the horses treated, including horses on stall rest, or horses showing a variety of anxious, aggressive, repetitive, and other problem behaviors [89]. It was generally well-tolerated, with reported side effects of sedation, decreased appetite or disinclination to eat medicated grain, and one case of urticaria that resolved following cessation of treatment [89]. Sedation associated with fluoxetine is typically mild, so that it should not impair ridden performance. However, as noted above, treated horses should be monitored for side effects, particularly at the start of treatment and following any dosage increases, and especially before attempting ridden work. In the study above, one horse was reported to experience profound sedation [89]. Conversely, although not described in horses, a recognized side effect of SSRIs in humans [91] and other animals is behavioral activation, which can manifest as increased agitation or irritability [91].

#### 10.4.3. Rapid-Onset, Short-Acting Medications

Many medications in this category produce significant effects on mentation, balance and coordination; thus, their use is not recommended during ridden work, and extra care should be taken when handling the horse on the ground. Some have significant analgesic effects, which may be beneficial in the context of retraining EGUS pain-related behaviors. Dosages of short-acting medications for use in facilitating behavior modification exercises should be selected with care to achieve anxiolysis without significant sedation.

##### Trazodone

Trazodone is a serotonin receptor antagonist and reuptake inhibitor (SARI) with a rapid onset of action, that can be used situationally or daily [92,93]. It should be administered 1 h prior to the behavior modification session, at a dosage of 2–8 mg/kg orally. Duration of effect is variable, reportedly ranging from 2–12 h, and dosing can be repeated q 12–24-h [93]. If given daily and long term, it should be tapered gradually, typically over a minimum of 2–4 weeks. An expected side effect with trazodone is mild sedation; pronounced sedation and ataxia, muscle fasciculations, transient tachycardias and arrhythmias, as well as soft stools and colic, have been reported at higher doses (7.5–10 mg/kg) [93]. Further, tolerance to its therapeutic effect can develop over time [93].

##### Alpha-2 Adrenergic Receptor Agonists

Drugs of this class, such as xylazine and detomidine, are traditionally used for equine sedation. However, they also have anxiolytic and analgesic properties, and several alpha-2 agonists are used at sub-sedative doses for their anxiety-relieving effects in dogs and cats [94,95]. Detomidine orotransmucosal gel is licensed for standing sedation in horses at a dose of 40 µg/kg, and has a duration of action of 90–180 min at this dose. It has shown promise in reducing equine fear associated with fireworks at a dose of 30 µg/kg [96]. The dose range for equine anxiolysis of this drug class that minimizes sedative effects, including ataxia, is yet to be defined for horses.

##### Gabapentinoids

Gabapentin is an anticonvulsant and analgesic medication used in neuropathic pain [97]. Although it is a structural analog of gamma-aminobutyric acid (GABA), it does not appear to bind to GABA receptors and its mechanism of action is currently incompletely understood. It is frequently used in dogs and cats for anxiolysis, and its use in equine neuropathic pain has been reported [98]. However, its oral bioavailability in the horse is poor, with commonly used doses unlikely to be efficacious [99]. A recent pharmacokinetic and pharmacodynamic study in horses examined higher doses (40–120 mg/kg) than have typically been used for clinical analgesia and anxiolysis (10–20 mg/kg), and found the higher doses to be well-tolerated [99]. Pregabalin has similar properties to gabapentin and appears to be more bioavailable in horses [100]. Appropriate dose ranges for the use of either gabapentin or pregabalin as an equine anxiolytic agent remain to be confirmed.

##### Benzodiazepines

The benzodiazepines are sedative-hypnotic drugs that exert their effects via the GABA_A_ receptor, by enhancing the inhibitory effects of GABA. They are used mostly to treat anxiety, panic disorders, seizures, and insomnia in people, as well as in dogs and cats. Although diazepam is widely used for equine anesthesia [101], there is little information on the use of this class of medications for treatment of fears and anxieties in the horse. A single case report describing treatment of foal rejection by a mare found alprazolam to promote calm behavior and reduce aggression, leading to eventual acceptance of nursing by the foal [102]. Known side effects of benzodiazepines include impaired memory and learning, paradoxical excitation, and disinhibition of aggression; these are serious considerations warranting caution in their use in conjunction with behavior modification for EGUS pain-related problem behaviors.

#### 10.4.4. Other Medications

Propanolol is a beta adrenoceptor blocker that is used in equine cardiology for treatment of supraventricular tachyarrhythmias. In humans, it is also used for anxiolysis and is known as the “stage fright drug” for its ability to blunt the effects of fear and anxiety by blocking norepinephrine receptors in the brain. Its pharmacokinetics in horses have been described [103], and it has been proposed for reducing equine fear-related behavior [104]. Anecdotally, one of the authors (BWS) has used it with some success at a dose of 0.3 mg/kg 30–45 min prior to exposure to a stressful event.

While other medications such as acepromazine, fluphenazine, and reserpine have been used to produce sedation and apparent “calming” in horses, they are not recommended for the purposes of anxiolysis in managing pain-related problem behaviors. Acepromazine, a phenothiazine antipsychotic commonly used as a tranquilizer, is no longer recommended for use as a behavior-modifying agent as it blunts behavior without providing a true anxiolytic effect [86]. Like acepromazine, fluphenazine is an antipsychotic and exerts its main effects via dopamine receptor antagonism; thus, it is not expected to produce significant anxiolysis. Reserpine is a plant-derived, long-acting sympatholytic alkaloid that depletes norepinephrine, serotonin, and dopamine. Although it has been commonly used historically in horses for its behavior-modifying effects, it is no longer recommended as it also suppresses behavior (including responses to external stimuli such as training cues) without true anxiolytic effects [86]. Furthermore, fluphenazine and reserpine both have narrow therapeutic windows and potential for severe side effects; an additional contraindication for reserpine in the current context is that it might promote gastric ulcer formation [105].

#### 10.4.5. Supplements and Alternative Therapies

The use of supplements and other non-pharmaceutical products might help to reduce anxiety and promote relaxation in some horses. Alpha-casozepine is a decapeptide tryptic hydrolysate of bovine milk α-_S1_ casein with structural similarities to benzodiazepines and effects via the GABA_A_ receptor [106]. It has demonstrated anxiolytic effects in stress in multiple species, without the tolerance and withdrawal effects associated with benzodiazepines, suggesting that its mechanism of action might involve a different subunit of GABA_A_ or a role of neurosteroids [107]. In horses, it has shown some benefit, namely improved tolerance, when given beginning 5 days prior to mildly aversive procedures [108], and in association with acclimation to a new environment and training for basic ground-handling procedures [109]. In contrast, there was no benefit in a study evaluating its potential benefits in trailer loading of horses [110].

Equine Appeasing Pheromone is a synthetic substance developed to mimic equine maternal pheromones, that is proposed to have a calming effect [111]. Results of research on this product have been mixed, with some benefits reported in adult horses during a novel object (fear) test [111], but no benefits found during weaning of foals [112] or during hoof trimming of adult horses [113]. Lavender oil has been used in other species for its calming properties; in horses it was found to decrease heart rate and salivary cortisol, and to improve behavioral markers of relaxation [114].

There has been increasing interest recently in the therapeutic use of cannabidiol (CBD) in humans and animals. This compound exerts its effects via the endocannabinoid system. There is some support for its use in fear and anxiety disorders, based on rodent and human research [115]. Limited research performed to date on this use in dogs has yielded mixed results with respect to its efficacy [116]. Preliminary research on this use of CBD in horses has also yielded mixed results [117,118]. There are no currently approved veterinary formulations, these products are often regulated, and in some jurisdictions, veterinarians cannot recommend or prescribe them to animals.

The equine supplement market comprises many products claiming to have a calming effect. The precise composition (ingredients, dose) and efficacy of these products is not closely regulated. An exhaustive examination of these products is beyond the scope of this commentary; however, it is important to consider the potential for adverse effects or interactions with medications, in addition to lack of efficacy, when administering supplements, and to examine the ingredient lists to this effect. For instance, while tryptophan is an amino acid precursor of serotonin and there is some support for its use in reducing anxiety, fear, and aggression, it may be associated with adverse effects in horses [119], and a number of studies evaluating it for a calmative effect have not found a behavioral benefit [120,121,122,123,124]. In addition, concurrent administration of tryptophan with serotonergic medications has the potential to increase the risk of serotonin syndrome.

## 11. Conclusions

Equine gastric ulcer syndrome is complex; a variety of physical and behavioral stressors can play a role in its development and influence how a horse’s gastric disease responds to medical treatment. Successful management of EGUS requires careful evaluation to ensure that other concurrent diseases are addressed, as well as analysis of husbandry and the environment to minimize the impact of associated stressors.

Pain-based behavior changes and problems commonly arise secondary to EGUS. These behavior changes can resolve readily with treatment of the underlying gastric disease, or in some cases problem behaviors that negatively affect interactions between horse and handler/rider and impair athletic function might persist because of learning. An approach to EGUS management that incorporates environmental and behavior modification, and appropriate use of behavior-modifying medications, in addition to addressing any concurrent diseases, has the potential to improve outcomes, particularly in chronic or refractory EGUS cases.

## 12. Future Directions

Pain remains challenging to assess and treat effectively in horses. It is not clear to what extent persistent behavior problems associated with EGUS result from the presence of persistent disease beyond endoscopic resolution vs. learned fear and anxiety vs. persistent pain states associated with abnormal pain processing resulting from EGUS vs. the impacts of other, concurrent, painful diseases.

Further, many horses are kept in environments that do not meet their behavioral needs. While providing freedom, access to forage, and social interaction with conspecifics is advisable, the specific manner in and degree to which they are provided, and how this relates to the presence or absence of EGUS and other stress-related health and behavioral problems, is not well characterized.

There is also a complex relationship between EGUS, sleep, and stress, that warrants further study. Finally, although there is increasing interest in the use of behavior-modifying medications in horses, relatively little research has been reported on the use of specific agents for anxiolysis. Given the recent rise in their use and their potential benefits and harms, it is imperative that research be conducted to explore appropriate dosing, safety, and efficacy of these drugs in the treatment of specific equine behavioral conditions.

## Figures and Tables

**Table 1:** Anxiolytic medications evaluated for use in horses.

Anxiolytic Medications Evaluated for Use in Horses, with Potential Benefits in EGUS									
Drug	Class	Dose	Time to Effect	Duration of Action	Treatment Duration	Weaning	Analgesia	Significant Sedation or Ataxia at Therapeutic Doses	Other
Fluoxetine	SSRI	0.15–0.5 mg/kg PO SID	4–6 weeks	Continuous use	Minimum 4–6 months, to lifelong	Yes, decrease dose in 25% increments, over 4–6 weeks or longer	No	Not expected	-
Trazodone	SARI	2–8 mg/kg PO PRN; or SID-BID	60 min	2–12 h	PRN	If given daily for more than 2 weeks	No	Yes	-
**Anxiolytic Medications of Potential Value in EGUS, for which Important Data is Lacking**									
Detomidine OTM gel	Alpha_2_	30 µg/kg OTM PRN reported for anxiolysis ^1^	40 min	90–180 min (40 µg/kg dose)	PRN	PRN	Yes	Yes	Wear gloves
Alprazolam	BZD	0.035–0.04 mg/kg up to TID reported for anxiolysis ^2^	unknown	unknown	PRN	If given daily for more than 2 weeks	Yes	Unknown	Controlled substance Can cause paradoxical excitation Can disinhibit aggression Amnestic
Gabapentin	GP	Information is largely anecdotal. Dosing, time to effect, and duration of effect to be Established.			PRN	If given daily for more than 2 weeks	Yes, neuropathic	Unknown	Controlled substance in some jurisdictions Poor oral bioavailability
Propranolol	Beta blocker				PRN	Likely indicated if given daily and long-term	No	Not expected	Cardiovascular effects?

Notes: ^1^ Detomidine OTM dose reported in one small study of horses exposed to fireworks; ^2^ Alprazolam dose reported in a single case study and a single pharmacokinetic study. Abbreviations: EGUS: equine gastric ulcer syndrome; SSRI: selective serotonin reuptake inhibitor; SARI: serotonin antagonist and reuptake inhibitor; OTM: orotransmucosal; Alpha_2_: alpha-2 adrenergic agonist; GP: gabapentinoid; BZD: benzodiazepine; beta blocker: beta adrenergic receptor antagonist.

## Data Availability

Not applicable as no new data was generated in this publication.

## Supplementary Materials

The following supporting information can be downloaded at: https://www.mdpi.com/article/10.3390/ani15203019/s1, Table S1: Example of a systematic desensitization and counterconditioning plan for a horse with “girthiness”; Table S2: Example of a retraining program for a behavior problem under saddle.
