# Supplementary material for: Incorporating a Behavioral Medicine Approach in the Multi-Modal Management of Chronic Equine Gastric Ulcer Syndrome (EGUS): A Clinical Commentary
Source: Animals (Basel). 2025 Oct 17;15(20):3019. doi: 10.3390/ani15203019 (PMC12561611; doi:10.3390/ani15203019)
Supplement: Supplementary file 1 [file animals-15-03019-s001.zip › animals-3860544-supplementary.pdf]

Incorporating a behavioral medicine approach in the multi-modal management of chronic equine gastric ulcer syndrome (EGUS): A clinical commentary.

Supplementary Material

Table S1. Example of a systematic desensitization and counterconditioning plan for a horse with "girthiness".

	Problem behavior: The horse pins its ears, swishes its tail, and will not stand still when approached closely with the saddle, while standing in the cross ties.
*All exercises performed starting on the horse's near (left) side	
Step 1	Separate the stimuli: choose a different location from the usual tacking-up site and separate the saddle from the girth and saddle pad(s). Avoid having any other associated riding equipment present.<br>Have an assistant stand with the horse on a loose lead line. The assistant should have an empty bucket and a supply of treats (*e.g.*, hay pellets) in a container that the assistant can reach easily but the horse can't access itself.<br>Begin each repetition when the horse is standing calmly without the need for restraint (loose lead line).
Step 2	Begin by standing far enough away from the horse that it sees you but shows no signs of tension, evasion, or aggression (*e.g.*, 3 meters). Stand facing the horse and holding the saddle pad down along the side of your body. Lift the pad slowly out to the side, and have the assistant tip a few hay pellets into the bucket. Lower the pad. Repeat, observing the horse's body language for signs of tension *vs.* relaxation. If the horse reacts with tension, evasion, or aggression, increase the distance and start over. If the horse remains calm, repeat several times.
Step 3	When the horse is consistently relaxed doing the above repetitions, decrease the distance slightly (*e.g.*, to 2.5 meters). Repeat the exercise in Step 2.<br>Each decrease in distance is a new step in the DS/CC process. If the horse remains relaxed, the distance can be gradually decreased over many repetitions, until the horse is relaxed with the person standing next to them on the near (left) side and lifting the saddle pad out to their side.
Step 4	Lift the pad and move it side to side and up and down (beginning with the smallest and slowest movements) without touching the horse, then have the assistant give pellets in the bucket and lower the arm with the pad. Repeat.<br>Gradually increase the size and speed of the movements, over many repetitions, until the horse is relaxed with the person standing next to them and moving the saddle pad around near them.
Step 5	As above, but touch the horse's shoulder with the saddle pad, give pellets, and lower the arm with the pad. Repeat.
Step 6	When the horse is consistently relaxed being touched with the saddle pad, progress to stroking the horse's shoulder with the pad. Give pellets while stroking with the pad and immediately lower the arm with the pad. Repeat.
Step 7	When the horse is consistently relaxed being stroked with the saddle pad, touch the pad to the shoulder, then slide the pad up the shoulder and onto the back (do not position it precisely). Give pellets, then remove the pad. Repeat.

Step 8	When the horse is consistently relaxed having the saddle pad put on his back, progress to touching the pad to the shoulder, sliding it up and putting it on the back, and positioning it in the correct location and configuration. Give pellets, remove pad. Repeat.
Step 9	When the horse is consistently relaxed having the saddle pad positioned on his back, progress to pressing on the pad in the areas where the saddle would press. Give pellets, remove pad. Repeat.
Step 10	When the horse is consistently relaxed having the saddle pad positioned on his back and pressed on, start over at Step 2, without the pad, but with the saddle only (no girth). Proceed through the steps described above, using both hands to hold and raise the saddle.
Step 11	When the horse is consistently relaxed when the saddle without a pad or girth is put on his back, begin the process again, at Step 2, with both the saddle pad and the saddle. Progress through the steps at a distance.
Step 12	When the horse is consistently relaxed with you standing next to it, holding and raising the saddle and pad, put the saddle down (*e.g.*, on a rack), and place the pad on his back, as before. Give pellets. Pick up the saddle, give pellets, and put the saddle down. Repeat.
Step 13	As above, but put the saddle pad on, and then progress through the steps used above to desensitize the horse up to the point of having the saddle put on its back.<br>The horse should be consistently relaxed at each step, before you proceed to the next step in training.
Step 14	Approach the horse with the pad and saddle, put the saddle down, and position the saddle pad, as above, then position the saddle on top. Give pellets, remove saddle and pad. Put the pad on again, then the saddle on, and give pellets. Remove both. Repeat.
Step 15	As above, but after putting the saddle on, lift the saddle flap, then give pellets, and step back. Repeat. Add in handling the billet/girth straps if the horse is relaxed, give pellets, release straps and flap, and step back. Repeat.
Step 16	As above, but walk to the other side of the horse, touch the saddle flap, give pellets, release the flap and step back. Repeat.
Step 17	As above, but add lifting the flap, then handling the billet straps. Give pellets, release the saddle flap and billet straps, and step back. Repeat.
Step 18	As above, but handle the saddle flaps and the billet straps on both sides, then give pellets, and step back. Repeat.
Step 19	Have a long girth nearby but not on the saddle. Put on the saddle pad and the saddle, as above. The assistant should be able to reach to steady or catch the saddle, if the horse moves suddenly. Standing several feet away, begin the process of presenting the girth at a distance (as done for the pad in Step 2, then the saddle, above). Repeat.<br>*Girth presentation and initial attachment performed on the horse's off (right) side
Step 20	Put on the saddle pad and the saddle and continue repeating the exercises where the girth is presented, incrementally decreasing your distance from the horse, if the horse remains relaxed, until you are standing next to the horse.
Step 21	Put on the saddle pad and the saddle from the near (left) side, give pellets, then move to the horse's off (right) side, and touch the horse's shoulder with the girth, give pellets, remove the girth and step back. Repeat.
Step 22	As above, and progress to putting the girth over the saddle, giving pellets, removing the girth and stepping back. Repeat.

Step 23	As above, and progress to handling the girth buckles while the girth is draped over the saddle. Give pellets, remove the girth, step back. Repeat.
Step 24	As above, and progress to lifting the saddle flaps and jingling the girth buckles. Give pellets, remove the girth, step back. Repeat.
Step 25	As above, and slide the girth off the saddle. Give pellets, step back. Repeat.
Step 26	As above, but add holding the girth hanging down from one hand and lifting the saddle flap. Give pellets, drop the flap and step back. Repeat
Step 27	As above, but add handling the billet straps while holding girth hanging down from one hand. Give pellets, drop the straps and the flap, and step back. Repeat.
Step 28	As above, but add attaching one girth strap to a billet strap. Give pellets, detach the girth and step back. Repeat.
Step 29	As above, but attach both girth straps on the same side of the horse. Give pellets, detach the girth, and step back. Repeat.
Step 30	As above, but let the girth hang and move it a little. Give pellets, detach the girth and step back. Repeat.
Step 31	As above, but let the girth hang and walk to the other side of the horse. Give pellets, walk back and detach the girth, and step back. Repeat.
Step 32	As above, and bend down a bit as if to reach for the girth. Give pellets, stand up, and step back (leave the girth attached). Repeat.
Step 33	As above, and reach your hand partway towards the girth (briefly). Give pellets, withdraw the hand, stand up, and step back. Repeat.
Step 34	As above, then reach for and grasp the girth (briefly). Give pellets, release the girth, and step back. Repeat.
Step 35	As above, but lift the girth towards the saddle without touching the horse's underside with it. Give pellets, release girth slowly and gently, and step back. Repeat.
Step 36	As above, and lift the girth enough for light contact with the horse's underside. Give pellets, release the girth slowly, and step back. Repeat.
Step 37	As above, and hold the girth lightly against the horse with one hand while lifting the saddle flap with the other. Give pellets, slowly and gently release the girth, and step back. Repeat.
Step 38	As above, and handle the billet straps. Give pellets, release the girth slowly and gently, and step back. Repeat.

Step 39	As above, and attach one girth strap very loosely. Give pellets, then detach the girth and release it slowly and gently, and step back. Repeat.
Step 40	As above, and attach both girth straps very loosely. Give pellets. Detach the girth, release it slowly and gently, and step back. Repeat.
Step 41	As above, and leave the girth very loosely attached. Give pellets. Lead the horse forward one step and give pellets. Leave the girth on and repeat these small leading movements, giving pellets each time.
Step 42	As above, but tighten the girth one more hole/inch, lead the horse forward a step as above, give pellets. Repeat.
Step 43	When the horse remains consistently relaxed, repeat the exercise in Step 42, gradually tightening the girth 1 extra hole/ at a time. Aim to do this over several sessions, until the horse remains calm as the girth is tightened enough to be able to mount and ride.

Notes: 1) The environment should be free of distractions (*e.g.*, other horses being turned out or fed) or anything that could startle or frighten the horse during training sessions. 2) Both the assistant and the trainer should observe the horse's body language for signs of tension, evasion, or aggression, and the assistant should alert the trainer immediately if they observe any such signs, so that the trainer can stop and move away. 3) Corrections (punishment) should not be used during training sessions. 4) Each step should be repeated several times before proceeding to the next step and increasing the difficulty. 5) Steps should not be omitted; it is better to reduce the number of repetitions, if all is going well, than to skip steps. 6) Training should be done over many sessions. Sessions should be kept short (*e.g*, no longer than 10 minutes), and should be ended on a successful note if possible, with a larger reward ("jackpot") followed by putting the food and horse away. 7) The horse should remain calm if training is proceeding at an appropriate pace. If the horse shows signs of tension, evasion, or aggression, go back one or more steps in the training process to where the horse is relaxed, and consider shortening the sessions and doing more repetitions at each step, before progressing to the next one.

Table S2. Example of a retraining program for a behavior problem under saddle.

	Problem behavior: The horse kicks out when given the aids for a trot transition from walk, when ridden in the arena. If stronger aids are applied, the horse bucks.<br><br>The horse knows how to be lunged and is familiar with verbal cues for walk and trot.
Step 1	Separate the stimuli: if possible, choose a different location from the usual riding area.<br>Have a supply of hay pellets in a wearable container easily accessed by the handler but not by the horse, and a small feed tub or bucket.
Step 2	Classically condition the secondary reinforcer: while standing next to the horse, holding the lead line (without tension), click and then drop some pellets into the feed tub. Allow the horse to eat them. Repeat several times (click, feed, eat).<br>Assess whether the secondary reinforcer has been classically conditioned (*i.e.*, does the horse know that it means food is coming): wait for the horse to look away from the feed tub/you, and then click, pause, and feed pellets. If the horse alerts (looks as though it expects food to appear in the tub) when it hears the click, the secondary reinforcer has been classically conditioned and is ready for use.
Step 3	Prepare to lunge the horse without a saddle, rider, or any restrictive gear. Have the wearable container of hay pellets, and the feed bucket nearby.<br>Ask the horse to walk forward on the lunge line. Click when it complies, ask the horse to stop, approach it and offer a few pellets in the tub. Repeat.
Step 4	Ask the horse to walk. After a few steps, click, ask it to stop, and approach to offer pellets in the tub. Repeat.<br>When the horse appears to understand the procedure (*e.g.*, slows or stops on its own, after the click), proceed to step 5.
Step 5	Ask the horse to walk forward and then give the verbal cue to trot. If the horse accelerates/looks as though it is about to trot, click, stop, approach, and offer pellets in the tub. Repeat.
Step 6	When the horse responds quickly to the verbal cue to trot, either by accelerating or by almost trotting, begin to select the best attempts (ideally, picking up the trot), still clicking right away, and giving pellets, almost every time (*e.g.*, at least 8 out of 10 tries). Do this for several repetitions.<br>When the horse picks up the trot 8 or more times out of 10 that the verbal cue is given, proceed to step 7.
Step 7	Ask the horse to walk forward, then give the verbal trot cue. As the horse completes one trot stride, click, stop, approach, and give hay pellets. Repeat several times.
Step 8	As above, but click after 2 trot strides. Stop, approach, and give hay pellets. Repeat.
Step 9	As above, but click after 3 trot strides. Stop, approach, and give hay pellets. Repeat.
Step 10	As above, but click after 4 trot strides. Stop, approach, and give hay pellets. Repeat.
Step 11	As above, but click after 5 trot strides. Stop, approach, and give hay pellets. Repeat.

Step 12	As above, but for the next several repetitions, vary the number of trot strides before the click, keeping the number between 2 and 7. Stop, approach, and give hay pellets. Give larger amounts of hay pellets for longer intervals of trot.
Step 13	As above, but for the next several repetitions, vary the number of trot strides before the click, keeping the number between 2 and 10. Stop, approach, and give hay pellets. Give larger amounts of hay pellets for longer intervals of trot.
Step 14	As above, but for the next several repetitions, vary the number of trot strides before the click, keeping the number between 2 and 15. Stop, approach, and give hay pellets. Give larger amounts of hay pellets for longer intervals of trot.
Step 15	As above, but for the next several repetitions, vary the number of trot strides before the click, keeping the number between 2 and 20. Stop, approach, and give hay pellets. Give larger amounts of hay pellets for longer intervals of trot.
Step 16	When the horse is easily making the trot transition and maintaining it, in the exercise above, start over at step 4, with a saddle and bridle on the horse. Proceed through all the steps up to step 15.
Step 17	When the horse is easily performing the exercises up to step 15, while wearing a saddle and bridle, start over at step 4, with a rider. The rider should not use the reins, nor give any other aids. It can be helpful to have the rider hold a neck strap or the saddle's pommel. Proceed through all the steps up to step 15.
Step 18	When the horse is easily performing the exercises up to step 15, while carrying a rider, start over at step 4, with the rider holding the reins. The rider should not give any aids. Proceed through all the steps up to step 15.
Step 19	When the horse is easily performing the exercises up to step 15, with a rider in the saddle and holding the reins, start over at step 4 and proceed to step 15, having the rider give very light aids after each verbal cue, just as the horse initiates the gait change.
Step 20	Next, have the rider very gradually move the application of light aids earlier and earlier in the sequence (*e.g.*, just after the verbal cue but before the horse starts to walk or trot forward, then at the same time as the verbal cue, and finally just before the verbal cue).
Step 21	When the horse is easily performing the exercises up to step 15 with a rider giving light aids just before the verbal cue, start over at step 4, having only the rider give light aids for the gait changes. If needed, the rider can give a verbal cue just after the leg aid. If needed after that, the person lunging can give a verbal cue. Proceed through the steps up to step 15.
Step 22	When the horse is easily performing the exercises up to step 15, with only the rider giving the aids, start over at step 4, but remove the lunge line. Have the person lunging remain in the middle of the circle and continue to have the horse move around that same circle. A verbal cue from the rider or the assistant can be added if needed. Repeat the steps through step 15.
Step 23	When the horse is easily performing the exercises up to step 15, with no lunge line and only the rider giving the aids, start over at step 4, but allow the horse to make a larger circle or oval, keeping the helper in the middle. If the horse does not respond to a leg aid for trot, at any time, the verbal cue

	can then be given by the rider, and if the horse still does not respond, the helper in the middle can give a verbal cue. Repeat the exercises through step 15.
Step 24	When the horse is easily performing the exercises up to step 15, with no lunge line and only the rider giving aids and on a larger circle/oval, gradually introduce going further and further away from the helper in the middle, still practising transitions. If the horse does not respond to a leg aid for trot, at any time, the verbal cue can then be given by the rider, and if the horse still does not respond, the helper in the middle can give a verbal cue. Continue to click, stop, and give pellets each time.
Step 25	When the horse is easily performing the trot transitions, with only the rider giving aids and going all the way out to the edges of the arena, on the track, gradually introduce changes of direction, continuing to practice the transitions. If the horse does not respond to a leg aid for trot, at any time, the verbal cue can then be given by the rider, and if the horse still does not respond, the helper in the middle can give a verbal cue. Continue to click, stop, and give pellets each time.
Step 26	When the horse is easily performing the trot transitions, with only the rider giving aids and riding everywhere in the arena, repeat the above step, without the helper present in the arena.<br>When the horse is easily performing the trot transitions, with only the rider giving aids and riding everywhere in the arena, without the helper present, proceed to step 27.
Step 27	Begin very gradually reducing the frequency of the food reinforcer, using a variable schedule of reinforcement. For this step, click, stop, and give pellets every 1-2 trot transitions. For example:<br>- Skip one trot transition, reinforce the next 2, then skip one, reinforce one, skip one, reinforce 3, skip one, reinforce one, skip one, etc.<br>Where possible, when no click and pellets are given, an alternate reinforcer, such as scratching the withers or verbal praise, should be given.
Step 28	As above, but click, stop, and give pellets every 1-3 trot transitions. Aim to always reward the best efforts by the horse, and to skip less well-executed transitions. For example:<br>- Skip 2 trot transitions, reinforce the next 3, skip one transition, reinforce the next 2, skip 2 transitions, reinforce the next one, skip one transition, reinforce the next one, etc.
Step 29	As above, but click, stop, and give pellets every 1-4 trot transitions. For example: Skip 3 trot transitions, reinforce the next 2, skip 2 transitions, reinforce the next 3, skip one transition, reinforce the next 2, skip 3 transitions, etc.
Step 30	As above, but click, stop, and give pellets every 1-5 trot transitions. For example:<br>- Skip 4 trot transitions, reinforce the next 4, skip 3 transitions, reinforce the next one, skip 2 transitions, reinforce the next 3, skip 4 transitions, reinforce 1, etc.
Step 31	As above, but click, stop, and give pellets every 1-7 trot transitions. For example:<br>- Skip 5 trot transitions, reinforce the next 2, skip 1 transition, reinforce the next 1, skip 4 transitions, reinforce the next 1, skip 6 transitions, reinforce the next 3, etc.
Step 32	As above, but click, stop, and give pellets every 1-10 trot transitions. For example:<br>- Skip 7 trot transitions, reinforce the next 3, skip 2 transitions, reinforce the next one, skip 5 transitions, reinforce the next 2, skip 8 transitions, reinforce the next 1, skip the next transition, reinforce the next one, etc.

Step 33	As above, but click, stop, and give pellets every 1-15 trot transitions. For example:<br>- Skip 10 transitions, reinforce the next 2, skip 1 transition, reinforce the next one, skip 5 transitions, reinforce the next 3, skip 11 transitions, reinforce the next one, skip 2 transitions, reinforce the next one, skip 12 transitions, reinforce the next one, etc.
Step 34	As above, but gradually further reduce the frequency, staying at a rate between 1-15 transitions, but reinforcing more often after longer intervals than after shorter ones. For example:<br>- Skip 10 trot transitions, reinforce one transition, skip 7 transitions, reinforce one, skip 13 transitions, reinforce one, skip 2 transitions, reinforce the next one, etc.
Step 35	When the horse is consistently performing trot transitions even at a low rate of reinforcement, it is possible to transition to using primarily non-food rewards, and only clicking, stopping, and giving pellets occasionally, for the best transitions.

Notes: 1) The environment should be free of distractions or anything that could startle or frighten the horse during training sessions. 2) The horse should remain calm if training is proceeding at an appropriate pace. If the horse shows signs of tension, evasion, or aggression, go back one or more steps in the training process to where the horse is relaxed. Do the same if the horse struggles to respond/does not respond to the cue. 3) Both the person lunging and the rider should monitor the horse's body language for signs of tension, etc.; such signs should be communicated immediately, so that the training session can be paused and restarted at an earlier step in the plan. 4) Corrections (punishment) should not be used during training sessions. 6) The aids should be released as soon as the horse performs the transition (negative reinforcement). 7) Each step should be repeated several times before proceeding to the next step step/increasing the difficulty. 8) Steps should not be omitted; it is better to reduce the number of repetitions, if all is going well, than to skip steps. 9) Training should be done over many sessions. Sessions should be kept short (*e.g.*, no longer than 10 minutes), and should be ended on a successful note, if possible, with a larger reward ("jackpot") followed by putting the food and the horse away.
